# Carbon Monoxide Attenuates Dextran Sulfate Sodium-Induced Colitis via Inhibition of GSK-3**β** Signaling

**DOI:** 10.1155/2013/210563

**Published:** 2013-11-14

**Authors:** Md. Jamal Uddin, Sun-oh Jeong, Min Zheng, Yingqing Chen, Gyeong Jae Cho, Hun Taeg Chung, Yeonsoo Joe

**Affiliations:** ^1^School of Biological Sciences, University of Ulsan, Ulsan 680-749, Republic of Korea; ^2^Department of Thoracic and Cardiovascular Surgery, Affiliated Hospital of Yanbian University, Yanji 133000, China; ^3^Department of Anatomy, School of Medicine, Institute of Health Sciences, Gyeongsang National University, Jinju 660-701, Republic of Korea

## Abstract

Endogenous carbon monoxide (CO) is produced by heme oxygenase-1 (HO)-1 which mediates the degradation of heme into CO, iron, and biliverdin. Also, CO ameliorates the human inflammatory bowel diseases and ulcerative colitis. However, the mechanism for the effect of CO on the inflammatory bowel disease has not yet been known. In this study, we showed that CO significantly increases survival percentage, body weight, colon length as well as histologic parameters in DSS-treated mice. In addition, CO inhalation significantly decreased DSS induced pro-inflammatory cytokines by inhibition of GSK-3**β** in mice model. To support the in vivo observation, TNF-**α**, iNOS and IL-10 after CO and LiCl treatment were measured in mesenteric lymph node cells (MLNs) and bone marrow-derived macrophages (BMMs) from DSS treated mice. In addition, we determined that CO potentially inhibited GSK-3**β** activation and decreased TNF-**α** and iNOS expression by inhibition of NF-**κ**B activation in LPS-stimulated U937 and MLN cells pretreated with CO. Together, our findings indicate that CO attenuates DSS-induced colitis via inhibition of GSK-3**β** signaling in vitro and in vivo. Importantly, this is the first report that investigated the molecular mechanisms mediated the novel effects of CO via inhibition GSK-3**β** in DSS-induced colitis model.

## 1. Introduction

Inflammatory bowel diseases (IBD) are a chronic and recurrent intestinal inflammation resulting from the transmural infiltration of neutrophils, macrophages, lymphocytes, and mast cells, ultimately giving rise to mucosal disruption and ulceration [1]. Furthermore, defects in epithelial barrier function and overproduction of proinflammatory cytokines such as IL-1*β*, IL-6, IL-12p40, IL-23p19, TNF-*α* and IFN-*γ* lead to tissue injury in intestine [2]. Additionally, upregulation of pro-inflammatory cytokines in IBD condition is mediated by NF-*κ*B, a transcription factor [3]. In order to develop the various models of experimental IBD, dextran sulfate sodium (DSS) or trinitrobenzene sulfonic acid (TNBS) was administrated [4, 5]. This model is characterized by acute tissue inflammation in the colon and mimics the pathology of human ulcerative colitis. 

Endogenous carbon monoxide (CO) as the end product of heme oxygenase-1 (HO-1) activity has anti-inflammatory, antiapoptotic and cytoprotective properties [6]. Also, CO ameliorates active inflammation in an experimental model of chronic IBD [7, 8]. Further, CO has showen beneficial effects in ischemia/reperfusion injury [9, 10], pulmonary inflammation [11], and sepsis [12]. In case of tracheal transplantation in mice, CO inhibits NF-*κ*B binding and iNOS expression [13]. In addition, CO-releasing molecules-2 (CORM)-2 which can deliver CO in the biological system have been found to reduce the inflammatory response by inhibition of NO and tumor necrosis factor (TNF-*α*) expression in mouse macrophages [14]. In particular, CORM-2 prevented the inflammation in murine colitis by inhibition of cytokine production [15]. Interestingly, recent studies have demonstrated that CO ameliorates active inflammation in an experimental model of chronic IBD or IL-10-deficient (−/−) mice, through induction of HO-1[7], but the precise mechanism remains unclear.

Glycogen synthase kinase-3 (GSK-3), a serine-threonine protein kinase, plays a vital role in glycogen metabolism, as well as regulation of cellular functions like control of cell division and apoptosis [16]. GSK3 is a constitutively active serine/threonine protein kinase having GSK-3*α* and GSK-3*β* isoforms. GSK-3*β* activity is inhibited by phosphorylation of serine 9 residue [17] and mediates the NF-*κ*B activity [18]. Also, selective inhibitors of GSK-3*β* were found to inhibit the inflammation and tissue injury due to downregulation of NF-*κ*B activity in acute colitis in rat [19]. On the one hand, GSK-3*β* expression was suppressed by HO-1 inducer hemin [20]. Therefore, CO as an end product of HO-1 catalytic reaction for breakdown of the heme moiety may inhibit the activation of GSK-3*β*.

The underlying mechanism of CO in the regulation of inflammatory response is not clear yet especially in DSS-induced colitis model. Therefore, we suggest that the therapeutic effects of CO on DSS-induced colitis result from the inhibition of GSK-3*β* and NF-*κ*B activation. 

## 2. Materials and Methods 

### 2.1. Reagents

Dextran sulfate sodium salt (DSS) was purchased from MP Biomedicals (LLC, France). Lipopolysaccharides (LPS), lithium chloride (LiCl), and tricarbonyldichlororuthenium (II) dimer (CORM2) were purchased from Sigma Aldrich (St. Louis, MO, USA). Phospho(p)-GSK-3*β* (serine9), GSK-3*β*, NOS2 (iNOS), p-IkB, and IkB were obtained from Santa Cruz Biotechnology (Santa Cruz, CA, USA). All other chemicals were obtained from Sigma-Aldrich.

### 2.2. Animals

Seven-week-old male C57BL/6 mice were obtained from ORIENT (Pusan, Korea). The mice were maintained in standard housing cages under specific pathogen-free conditions at 22°C and access to drinking water *ad libitum*. Mice were allowed to drink with or without 3% DSS water for 6 days and then mice were inhaled with or without CO (250 ppm) for 4 h or injected with LiCl (200 mg/kg, i.p.) on daily basis for 10 or 12 days. All mice were being fed with standard laboratory chow *ad libitum* at all times. Control mice were given only water. Water and chow consumption was comparable between DSS and control groups, both before and during the induction of colitis. Body weight was recorded daily and survival percent was monitored at 10 and 12 days respectively. After 10 days of CO or LiCl treatment, mice were sacrificed and colons from all mice were collected for histological and molecular assessment of inflammation. Experiments with mice were approved by the Animal Care Committee of the University of Ulsan.

### 2.3. Isolation and Culture of Bone Marrow Macrophages (BMMs) and Mesenteric Lymph Node Cells (MLNs)

Six- to 7-week-old C57BL/6 mice were provided with or without 3% DSS water for 6 days. BMMs were isolated as previously described [21]. After sacrificing the mice, femora and tibiae were carefully taken out and dissected free of adherent soft tissue. Bone marrow cells were collected by flushing the cavity by slowly injecting MEM-*α* medium (Hyclone, Loan, UT, USA). Cells were washed with PBS twice, and then the cells were taken in MEM-*α* medium containing 10% FBS, 50 units/mL penicillin, 50 *μ*g/mL streptomycin (Gibco, Grand Island, NY, USA). Cells were cultured in 10 cm tissue culture dishes at an amount of 2 × 10^6^ cells/dish and mouse macrophage colony stimulating factor (M-CSF, 10 ng/mL, BioSource, Camarillo, CA, USA) was added to differentiate BMMs. Three days later, nonadherent cells were removed and adherent cells (immature BMM) were suspended in fresh MEM-*α* with M-CSF and used for experiment. On the other hand, mesenteric lymph nodes were also isolated from mice treated with or without 3% DSS and MLNs were pressed through a cell strainer (Falcon 2340; BD Biosciences, San Jose, CA, USA) to get single cells. Cells were collected on DMEM containing 10% FBS and antibiotics. After washing with medium, cells were counted and used for subsequent experiment. Cells were treated with CORM2 and LiCl and then stimulated with or without LPS (1 *μ*g/mL) for designated time points. 

### 2.4. Cell Culture

U937 cells were cultured in DMEM in addition to 10% FBS and 1% penicillin streptomycin at 37°C in 5% CO_2_ until 75–80% confluence. After that, cells at the rate of 5 × 10^5^/mL were splited in 6-well plates and incubated for 18 h. Then cells were pretreated with CORM2 or LiCl and then treated with or without LPS (1 *μ*g/mL) for 24 h. After incubation, cells were harvested for western blotting and RT-PCR, and supernatant was collected to perform ELISA assay (R&D systems, Inc., Minneapolis, MN, USA) for measuring the level of TNF-*α* production as well.

### 2.5. Histological Analysis

After sacrificing the mice, the entire colon was dissected and flushed with ice-cold PBS. For histological analysis, mice colons were fixed in 10% neutral-buffered formalin for 24 h at room temperature, and paraffin-embedded tissue sections were stained with HE (hematoxylin and eosin) using standard techniques. 

### 2.6. Western Blotting

Colon tissue or cell extracts were prepared using lysis buffer containing RIPA buffer, protease inhibitor, and phosphatase inhibitors. Protein concentration in the lysate was measured by BCA assay (Pierce Biotechnology Inc., Rockford, IL, USA). An equal amount of protein was subjected to electrophoresis and then proteins were transferred to polyvinylidene difluoride (PVDF) membrane. After transfer, the membranes were blocked with 5% nonfat milk in PBS containing 0.1% Tween 20 (PBS-T) for 20 min and incubated at 4°C overnight with primary antibodies and followed by secondary antibodies conjugated with horseradish peroxidase for pGSK-3*β*, GSK-3*β*, pI*κ*B, I*κ*B, iNOS and *β*-actin. Enhanced chemiluminescence (ECL) western blotting detection system (GE Healthcare Life Sciences, Buckinghamshire, UK) was used to visualize the protein bands.

### 2.7. Reverse Transcription-Polymerase Chain Reaction (RT-PCR)

Total RNA was extracted from colon tissue or cell pellet using TRIzol reagent (Invitrogen, CA, USA) according to the manufacturer's instructions. In short, 2 *μ*g of total RNA was used to make cDNA by using M-MLV reverse transcriptase (Promega Corporation, WI, USA) and oligo (dT) 15 primer (Promega Corporation, WI, USA). The resulted cDNA was subjected to PCR for mouse GAPDH (f-aggccggtgctgagtatgtc, r-tgcctgcttcaccttct, 530 bp), 18S (f-cagtgaaactgcgaatggct, r-tgccttccttggatgtggta, 397 bp), iNOS (f-ccaccttggtgaagggactgagct, r-gctgcggggagccattttggt, 381 bp), TNF-*α* (f-agcccacgtcgtagcaaaccaccaa, r-acacccattcccttcacagagcaat, 421 bp) and IL-10 (f-gacaataactgcacccactt, r-tcaaatgctccttgatttct, 250 bp); and human GAPDH (f-ccacccatggcaaattccatggca, r-tctagacggcaggtcaggtccacc, 520 bp), iNOS (f-cagtacgtttggcaatggagactgc, r-ggtcacattggaggtgtagagcttg, 340 bp), t-bet (f-gctgtgcaggtgttgagcc, r-cataactgtgttcccgaggtgtc), and GATA-3 (f-gcctgtgcaaaagagatttcagat, r-tgattcacagagcatgtaggcc). GAPDH or 18S was used as internal loading control. The PCR products were detected on 2% agarose gels using digital gel documentation set.

### 2.8. Enzyme-Linked Immunosorbent Assay (ELISA)

U937 and MLN cells were incubated overnight on 6-well plate and then pretreated with CORM2 and LiCl for 30 min followed by stimulated with LPS (1 *μ*g/mL) for 24 h. Supernatant was collected from different experimental samples and levels of TNF-*α* were assayed by using human ELISA kit (BD Biosciences, San Diego, CA, USA) in U937 cells and mouse ELISA kit (R&D systems) in MLN cells.

### 2.9. Statistical Analysis

Results are expressed as the means ± SD. Statistical analysis was performed with the GraphPad Prism software version 5 (GraphPad Software Inc., San Diego, CA, USA). Differences in the data among the groups were analyzed using one-way ANOVA followed by Tukey's post hoc test. 

## 3. Results

### 3.1. CO Ameliorates Survival, Body Weight, and Colon Length in DSS-Induced Colitis

**Figure 1:**
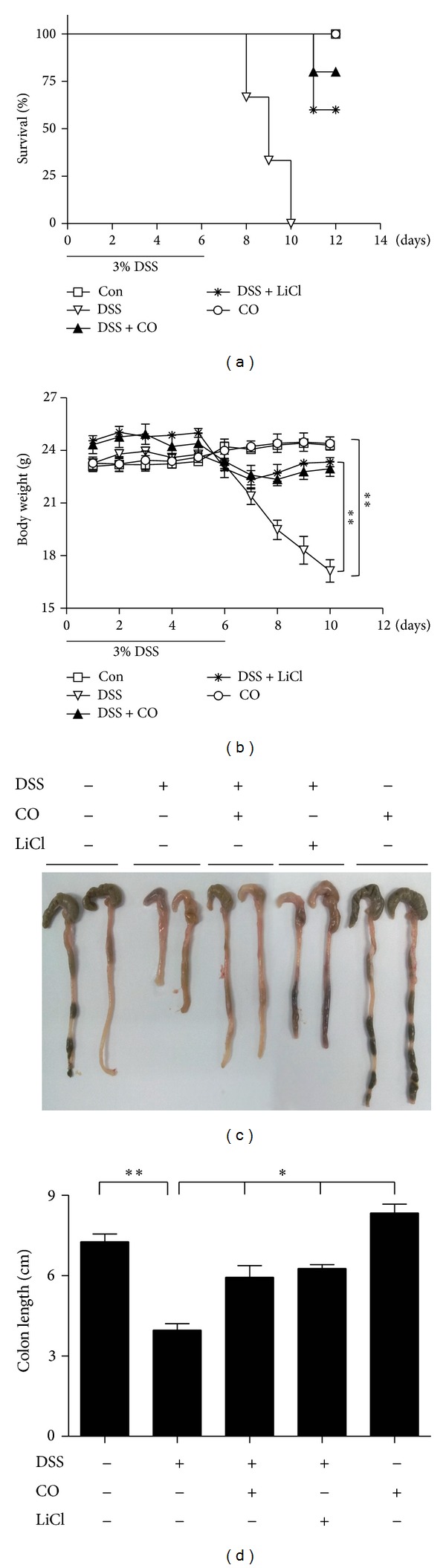
CO attenuates DSS-induced experimental colitis as measured by survival, body weight, and colon length. Mice were administrated with 3% DSS with drinking water for 6 days and CO (250 ppm) inhalation for 4 h daily and injection of LiCl (200 mg/kg, i.p) was performed daily for more 4 or 6 days. (a) Survival percent was measured at day 12. (b) Body weight was measured at day 10. (c) and (d) Colon length. (c) Representative images of 3 tests conducted in each group. (d) Data are mean ± SD for 3 mice. Con: Control, DSS: dextran sufate sodium salt and Data represents mean ± SEM, *P < 0.05, **P < 0.01.

In this study, colitis was induced by providing mice with 3% DSS water for 6 days. To examine the in vivo effects of CO or GSK-3*β* inhibitor (LiCl) on survival of DSS-induced colitis mice, we inhaled mice with CO (250 ppm) for 4 h or administrated LiCl (200 mg/kg, i.p.) on daily basis to mice for more 6 days. We found that mice from DSS group had 0% survival rate after 11 days of DSS treatment. On the other hand, the mice treated with CO or LiCl in the presence of the DSS had significantly higher rate of survival compared to DSS group (Figure 1(a)). Similarly, to determine the in vivo effects of CO or GSK-3*β* inhibitor (LiCl) on body weight of DSS-induced colitis mice, CO (250 ppm) inhalation for 4 h or administrated LiCl (200 mg/kg, i.p.) on daily basis was performed. Interestingly, we found those mice treated with CO or LiCl in the presense of the DSS had significantly higher body weight compared to that of DSS group (Figure 1(b)). According to the data, preventive treatment with CO is capable of increasing the survival rate as well as body weight of mice with colitis induced by DSS. At prescheduled time points, mice were killed, and the entire colon of mice were taken and then imaging was performed using a light imaging box and colon length was determined using a measuring scale. We found that DSS induced colitis significantly shortened colon length and, inhalation of CO or LiCl administration significantly recovered DSS effects (Figures 1(c) and 1(d)), suggesting beneficial effects of CO on DSS-induced colitis where inhibition of GSK-3*β* is involved.

### 3.2. CO Attenuates Experimental Colitis as Measured by Histology and Inflammatory Cytokines in Colon

**Figure 2:**
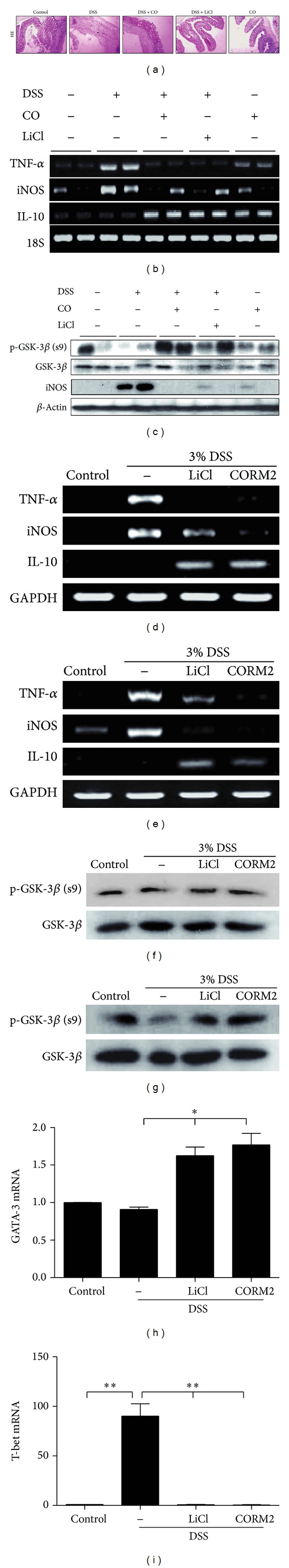
CO attenuates experimental colitis as measured by histology in colon and inflammatory cytokines in colon, MLNs, and BMMs. Mice were administrated with 3% DSS with drinking water for 6 days and CO (250 ppm) inhalation for 4 h daily and injection of LiCl (200 mg/kg, i.p) was performed daily for more 4 days. (a) Colon sections were subjected to H&E staining. (b) TNF-*α*, iNOS, and IL-10 mRNA levels were detected from colon tissue by RT-PCR. (c) iNOS and pGSK-3*β* protein levels were measured from colon tissue by western blotting. To detect the levels of cytokines in MLNs and BMM cells, mice were treated with 3% DSS solution for 6 days and isolated MLN and BMM cells were treated with CORM2 (100 *μ*M) or LiCl (20 mM) for 6 h. The levels of TNF-*α*, iNOS, and IL-10 mRNA were performed by using RT-PCR in MLN cells (d) and BMMs (e) and the levels of pGSK-3*β* were detected with western blot in MLN (f) and BMM (g) cells. Also, mRNA levels of GATA-3 (h) and t-bet (i) were performed by using real-time RT-PCR in MLN cells. Data represents mean ± SEM, *P < 0.05, **P < 0.01, and ***P < 0.001.

To know the effect of CO on GSK-3*β* and DSS-induced colitis, we inhaled mice with CO (250 ppm) for 4 h or administrated LiCl (200 mg/kg, i.p.) on daily basis to mice for more 4 days. Mice were sacrificed at designated time points; the entire colons were dissected and fixed in 10% formalin. We have characterized the histological features of paraffin-embedded tissue sections from mice colons by H&E staining. The histological features of the colons of CO treated and LiCl treated group were better than those of DSS-treated group (Figure 2(a)). To confirm the histological results, we checked mRNA expression patterns of TNF-*α*, iNOS and IL-10 in colon tissue obtained from DSS induced colitis mice following administration of CO and LiCl. Interestingly, CO or LiCl was found to be involved with significant suppression of TNF-*α*, iNOS and at the same time IL-10 mRNA level was found to significantly increase in colon tissue in vivo (Figure 2(b)). To further confirm the results, expression pattern of pGSK-3*β* and iNOS protein in colon tissue obtained from DSS-induced colitis mice following administration of CO and LiCl was examined. We found that CO or LiCl significantly increased expression of pGSK-3*β* and at the same time iNOS protein was found to decrease in colon tissue in vivo (Figure 2(c)). Results indicating the role of CO in inhibition of DSS-induced colitis are mediated through inhibition of GSK-3*β* activation.

### 3.3. CO Regulates the Production of Cytokines in MLNs and BMMs from DSS-Induced Colitis

Colitis was induced to mice through providing 3% DSS in drinking water for 6 days. After 6 days, mice were sacrificed to collect mesenteric lymph node and bone marrow. To investigate the effect of CO or LiCl on colitis-induced inflammatory cytokines, MLNs and BMMs were treated with CORM2 (100 *μ*M) or LiCl (20 mM) for 6 h and harvested cells were subjected for mRNA analysis by RT-PCR. Data showed that CORM2 and GSK-3*β* inhibitor significantly downregulated DSS induced proinflammatory cytokines such as TNF-*α* and iNOS mRNA level by increasing mRNA level of IL-10 in MLNs (Figure 2(d)) and BMMs (Figure 2(e)) respectively. To confirm the role of GSK-3 signaling in CORM2 or LiCl-treated MLNs and BMMs, we detected the levels of pGSK-3*β* using western blot analysis. The expression of pGSK-3*β* inhibited by DSS treatment was increased with CORM2 or LiCl in MLNs (Figure 2(f)) and BMMs (Figure 2(g)). Also, according to the report of Marques et al. [22], HO-1/CO system could regulate the Th1/Th2 profile. To investigate the association between GSK-3*β* signaling and Th1/Th2 profiling, we analyzed the levels of GATA-3 for Th2 cells and t-bet for Th1 cells using real-time PCR analysis. 

The levels of GATA-3 were increased by LiCl or CORM2 treatment (Figure 2(h)). DSS-induced t-bet levels were suppressed by CORM2 or LiCl treatment (Figure 2(i)). Therefore, these data suggested that CO mediates inhibition of colitis-induced proinflammatory cytokines and regulation of Th1/Th2 profile via inhibition of GSK-3*β* activation.

### 3.4. CO Controls GSK-3*β* Signaling in Human Macrophage Cell Lines and Mesenteric Lymph Node Cells (MLNs)

**Figure 3:**
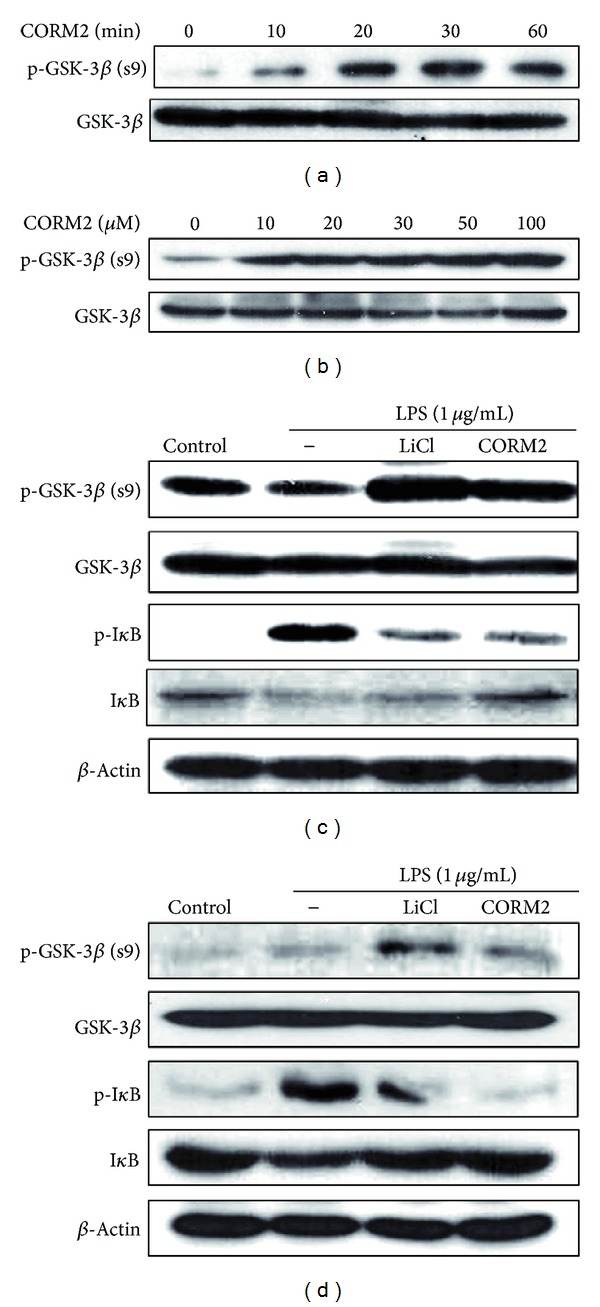
CO attenuates expression of GSK-3*β* signaling in human macrophage cell lines and MLNs. (a) U937cells were incubated with CORM2 (100 *μ*M) for various time periods (0, 10, 20, 30, and 60 min) (b) Cells were treated with CORM2 in a dose dependent manner (0, 10, 20, 30, 50, and 100*μ*M) for 30 min. (c) U937cells were preincubated with CORM2 (100 *μ*M) and LiCl (20 mM) for 30 min and then stimulated with LPS (1 *μ*g/mL) for 30 min. (d) MLNcells were preincubated with CORM2 (100 *μ*M) and LiCl (20 mM) for 30 min and then stimulated with LPS (1 *μ*g/mL) for 30 min. Protein expressions of pGSK-3*β* and pI*κ*B were detected by western blotting.

GSK3 is a constitutively active serine/threonine protein kinase having GSK-3*α* and GSK-3*β* isoforms. GSK-3*β* activity is tightly controlled by phosphorylation of regulatory serine 9 leading to its inhibition [17]. To monitor the CO effects on GSK-3*β* signaling, U937 cells were time and dose dependently treated with CORM2 and western blotting was performed. CORM2 strongly increased the pGSK-3*β* in the time-and dose dependent manners (Figures 3(a) and 3(b)). To confirm the in vivo observation of whether CO effect is mediated through inhibition of GSk-3*β*, U937 monocytes were pretreated with CORM2 (100 *μ*M) and LiCl (20 mM) for 30 min and then cells were stimulated with LPS (1 *μ*g/mL) for 30 min and harvested cells were subjected to protein analysis for inactive form of GSK-3*β* and activity of NF-*κ*B. Interestingly, we observed that LPS mediated activation of GSK-3*β* was inhibited with treatment of CORM2 or LiCl by increasing pGSK-3*β* expression (Figure 3(c)). The influence of GSK-3*β* inhibition on the activities of transcription factors, NF-*κ*B, was assessed, as it is known to regulate cytokine-mediated inflammatory responses [23]. Further, LPS increased NF-*κ*B activity downstream of GSK-3*β* by I*κ*B activation and CORM2 and LiCl significantly abrogated LPS effects (Figure 3(c)). To confirm the above and gain insight whether GSK-3*β* inhibition directly impairs the function of intestinal immune cells, in vitro stimulation experiments were performed with MLN cells. Similarly, we found that LPS stimulated GSK-3*β* and NF-*κ*B activation was potentially reduced by CORM2 as well as GSK-3*β* inhibitor in MLN cells (Figure 3(d)). The data presents that anti-inflammatory effect of CO is mediated by inhibition of GSK-3*β* and its downstream NF-*κ*B activation.

### 3.5. CO Downregulates TNF-*α* and iNOS Expression via Inhibition of GSK-3*β* Signaling

**Figure 4:**
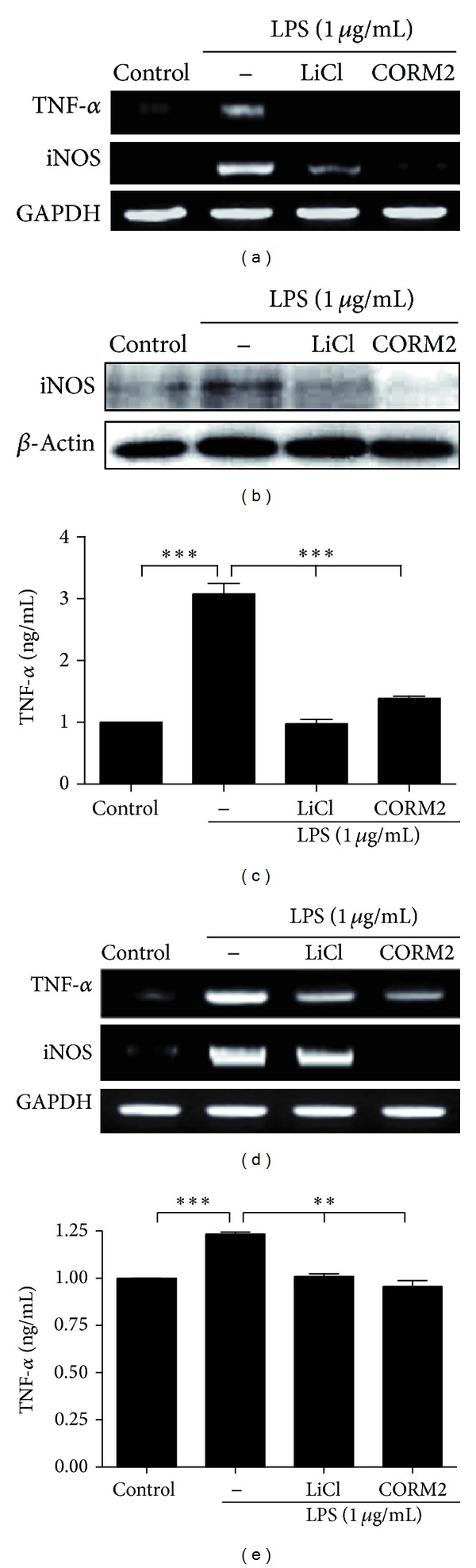
CO downregulates TNF-*α* and iNOS expression via inhibition of GSK-3*β* signaling. (a) to (c) U937 cells were pretreated with CORM2 (100 *μ*M) and LiCl (20 mM) for 30 min followed by stimulation with LPS (1 *μ*g/mL) for 24 h. (a) TNF*α* and iNOS mRNA expression was measured by RT-PCR, (b) iNOS protein expression was measured by western blotting, and (c) supernatant was collected and TNF-*α* protein level was measured by ELISA. (d) to (e) MLN cells were pre-incubated with CORM2 (100 *μ*M) and LiCl (20 mM) for 30 min and then stimulated with LPS (1 *μ*g/mL) for 24 h. (d) iNOS and TNF-*α* mRNA expression was measured by RT-PCR, and (e) Supernatant was used to measure TNF-*α* protein level by ELISA. The representative band or blot is shown. Data represents mean ± SEM, *P < 0.05, **P < 0.01, ***P < 0.001.

GSK-3*β* positively regulates the most important transcription factor in immune system NF-*κ*B, which controls proinflammatory responses [24, 25]. To obtain insight into the underlying mechanism responsible for inhibition of inflammatory effect of GSK-3*β* by CO in vitro, we pretreated U937 monocytes with CORM2 (100 *μ*M) and LiCl (20 mM) for 30 min, and then cells were stimulated with LPS (1 *μ*g/mL) for 24 h and harvested cells were subjected to protein and mRNA analysis of TNF-*α* and iNOS. Data showed that CORM2 and LiCl significantly decreased LPS induced TNF-*α* and iNOS expression (Figures 4(a), 4(b), and 4(c)). To confirm CO effects on pro-inflammatory cytokines in relation to GSK-3*β*, we used MLN cells and pretreated them with CORM2 (100 *μ*M) or LiCl (20 mM) for 30 min and then cells were incubated with LPS (1 *μ*g/mL) for 24 h. Likewise, CORM2 and LiCl significantly decreased LPS-induced TNF-*α* and iNOS expression in MLN cells as well (Figures 4(d) and 4(e)). Results from U937 and intestinal immune cells indicate that anti-inflammatory effects of CO took place by inhibition of GSK-3*β*.

## 4. Discussion

Underlying mechanism behind the pathogenesis of the human IBD is very complex due to the involvement of various factors like genetic, immunologic, and environmental factors [26, 27]. Recently, carbon monoxide (CO) is well known to have various functions in immunomodulation, anti-inflammation, and physiological homeostasis [28]. In addition, CO was found to have protective effects against ulcerative colitis [7] and chronic intestinal inflammation [29], respectively. Exogenous CO provides the anti-inflammatory response in case of hyperoxia condition [14], organ transplantation [30], and ischemia reperfusion (I/R) injury [31, 32]. Furthermore, the protective effects of CO have been investigated in intestinal inflammation both in vitro and in vivo. Thus, CO can be a potential therapeutic option in case of IBD due to its effectiveness in alleviating intestinal inflammation and augment mucosal compensation [33, 34]. However, molecular mechanism behind the beneficial effects of CO in intestinal inflammation is not yet clear. In this study, we showed that DSS-induced colitis is attenuated by CO-mediated inhibition of GSK-3*β* signaling in experimental mice model.

GSK-3*β* is a constitutively active serine/threonine protein kinase that is involved in a large number of cellular functions [17] and is capable of regulating the activity of NF-*κ*B, a key transcription factor for proinflammatory immune responses [35]. Inhibition of GSK-3*β* signaling has been reported to reduce experimental colitis in rat [19]. Increased GSK-3*β* activity was found to decrease IL-10 production leading to dampen inflammatory processes in intestinal immune cells [36] and macrophages [37]. Further, rat model of colitis exhibits many of the macroscopic, histological, and immunological features of inflammatory bowel disease (IBD) with neutrophilic involvement [19]. In our study, we found that CO and GSK-3*β* inhibitor significantly improved the survival of DSS-treated mice by increasing body weight, colon length and histological parameters, suggesting that CO inhalation improved experimental colitis by inhibition of GSK-3*β* signaling. However, DSS- [38] and TNBS- [39, 40] induced colitis have been found to be increased in colonic TNF-*α* levels. Further, CO ameliorated colitis in IL-10−/− [41] and TCR*α*−/− mice and therapeutic effects of CO correlated with induction of IL-10 [8, 42]. In the present study, CO and LiCl significantly decreased DSS-induced TNF-*α* and iNOS and in the same time inactive form of GSK-3*β* increased in colonic tissues in mice in vivo. Additionally, ex vivo experiment showed that DSS-induced TNF-*α* and iNOS expression was reduced by CORM2 and LiCl by increasing IL-10 in MLNs and BMMs. Results from in vivo and ex vivo experiments indicate regulation of GSK-3*β* by CO in DSS-induced experimental mice model. 

GSK-3*β* inhibitors reduced the systemic inflammatory response, tissue injury, and the phosphorylation of NF-*κ*B and its downstream genes in the lung tissue of rats [43]. To confirm the in vivo data regarding regulation of colitis and involvement of CO and GSK-3*β*, we did some in vitro experiments with U937 monocytes and MLN cells. CORM2 potentially increased the pGSK-3*β* with time- and dose-dependent manners. In addition, CORM2 and LiCl significantly decreased LPS-induced NF-*κ*B activation by increasing I*κ*B and pGSK-3*β*(ser9) in U937 as well as MLN cells as described [44]. 

Proinflammatory cytokines play an important role in the inflammatory events in IBD. Blockade of GSK-3*β* attenuates TLR-mediated excessive proinflammatory cytokines and constitutes a promising therapeutic option for reducing intestinal immune reactions toward the luminal flora in inflammatory bowel disease [45]. In our observation, it is provided that TLR4 ligand, and LPS-induced inflammatory responses (TNF-*α*, iNOS) were downregulated by CORM2 and LiCl treatment in U937 and MLN cells. Data presents that CO inhibits inflammatory cytokines induced by LPS via inhibition of GSK-3*β* signaling in vitro as well. 

To our knowledge, these results are the first to characterize anti-inflammatory properties of CO via inhibition of GSK-3*β* in a DSS-mediated model of chronic colonic inflammation. The anti-inflammatory effects of CO are attributed to the inhibition of GSK-3*β* signaling and highlight the broad impact of these pathways on intestinal inflammation. GSK-3*β* inhibition correlated with increased IL-10 expression, which may be relevant anti-inflammatory mechanism of this pathway, because IL-10 was reported to have a protective role in colonic inflammation [42]. Also, the balance of cytokines production is controlled with the regulation of Th1 and Th2 profile by HO-1/CO system [22]. In summary, our data demonstrate that CO decreases inflammatory responses via inhibition of GSK-3*β* and pro-inflammatory cytokines in DSS-induced colitis. Our mechanism provided in the CO-induced protection against DSS-induced colitis might play an important role in the treatment of ulcerative colitis.

## References

[1] Fiocchi C (1998). Inflammatory bowel disease: etiology and pathogenesis. *Gastroenterology*.

[2] Xavier RJ, Podolsky DK (2007). Unravelling the pathogenesis of inflammatory bowel disease. *Nature*.

[3] Lawrence T, Bebien M, Liu GY, Nizet V, Karin M (2005). IKK*α* limits macrophage NF-*κ*B activation and contributes to the resolution of inflammation. *Nature*.

[4] Okayasu I, Hatakeyama S, Yamada M, Ohkusa T, Inagaki Y, Nakaya R (1990). A novel method in the induction of reliable experimental acute and chronic ulcerative colitis in mice. *Gastroenterology*.

[5] Yamada Y, Marshall S, Specian RD, Grisham MB (1992). A comparative analysis of two models of colitis in rats. *Gastroenterology*.

[6] Ryter SW, Morse D, Choi AMK (2007). Carbon monoxide and bilirubin: potential therapies for pulmonary/vascular injury and disease. *American Journal of Respiratory Cell and Molecular Biology*.

[7] Hegazi RAF, Rao KN, Mayle A, Sepulveda AR, Otterbein LE, Plevy SE (2005). Carbon monoxide ameliorates chronic murine colitis through a heme oxygenase 1-dependent pathway. *The Journal of Experimental Medicine*.

[8] Sheikh SZ, Hegazi RA, Kobayashi T (2011). An anti-inflammatory role for carbon monoxide and heme oxygenase-1 in chronic Th2-mediated murine colitis. *Journal of Immunology*.

[9] Nakao A, Toyokawa H, Abe M (2006). Heart allograft protection with low-dose carbon monoxide inhalation: effects on inflammatory mediators and alloreactive T-cell responses. *Transplantation*.

[10] Kohmoto J, Nakao A, Stolz DB (2007). Carbon monoxide protects rat lung transplants from ischemia-reperfusion injury via a mechanism involving p38 MAPK pathway. *American Journal of Transplantation*.

[11] Goebel U, Siepe M, Mecklenburg A (2008). Carbon monoxide inhalation reduces pulmonary inflammatory response during cardiopulmonary bypass in pigs. *Anesthesiology*.

[12] Cepinskas G, Katada K, Bihari A, Potter RF (2008). Carbon monoxide liberated from carbon monoxide-releasing molecule CORM-2 attenuates inflammation in the liver of septic mice. *American Journal of Physiology: Gastrointestinal and Liver Physiology*.

[13] Minamino T, Christou H, Hsieh C-M (2001). Targeted expression of heme oxygenase-1 prevents the pulmonary inflammatory and vascular responses to hypoxia. *Proceedings of the National Academy of Sciences of the United States of America*.

[14] Sawle P, Foresti R, Mann BE, Johnson TR, Green CJ, Motterlini R (2005). Carbon monoxide-releasing molecules (CO-RMs) attenuate the inflammatory response elicited by lipopolysaccharide in RAW264.7 murine macrophages. *The British Journal of Pharmacology*.

[15] Takagi T, Naito Y, Uchiyama K (2011). Carbon monoxide liberated from carbon monoxide-releasing molecule exerts an anti-inflammatory effect on dextran sulfate sodium-induced colitis in mice. *Digestive Diseases and Sciences*.

[16] Ali A, Hoeflich KP, Woodgett JR (2001). Glycogen synthase kinase-3: properties, functions, and regulation. *Chemical Reviews*.

[17] Jope RS, Johnson GVW (2004). The glamour and gloom of glycogen synthase kinase-3. *Trends in Biochemical Sciences*.

[18] Frame S, Cohen P (2001). GSK3 takes centre stage more than 20 years after its discovery. *The Biochemical Journal*.

[19] Whittle BJR, Varga C, Pósa A, Molnár A, Collin M, Thiemermann C (2006). Reduction of experimental colitis in the rat by inhibitors of glycogen synthase kinase-3*β*. *British Journal of Pharmacology*.

[20] Ndisang JF, Lane N, Jadhav A (2009). Upregulation of the heme oxygenase system ameliorates postprandial and fasting hyperglycemia in type 2 diabetes. *American Journal of Physiology: Endocrinology and Metabolism*.

[21] Chambers TJ, Owens JM, Hattersley G, Jat PS, Noble MD (1993). Generation of osteoclast-inductive and osteoclastogenic cell lines from the H-2KbtsA58 transgenic mouse. *Proceedings of the National Academy of Sciences of the United States of America*.

[22] Marques VP, Gonçalves GM, Feitoza CQ (2006). Influence of TH1/TH2 switched immune response on renal ischemia-reperfusion injury. *Nephron Experimental Nephrology*.

[23] Blackwell TS, Christman JW (1997). The role of nuclear factor-*κ*B in cytokine gene regulation. *American Journal of Respiratory Cell and Molecular Biology*.

[24] Martin M, Rehani K, Jope RS, Michalek SM (2005). Toll-like receptor-mediated cytokine production is differentially regulated by glycogen synthase kinase 3. *Nature Immunology*.

[25] Demarchi F, Bertoli C, Sandy P, Schneider C (2003). Glycogen synthase kinase-3*β* regulates NF-*κ*B1/p105 stability. * The Journal of Biological Chemistry*.

[26] Badger AM, Bradbeer JN, Votta B, Lee JC, Adams JL, Griswold DE (1996). Pharmacological profile of SB 203580, a selective inhibitor of cytokine suppressive binding protein/p38 kinase, in animal models of arthritis, bone resorption, endotoxin shock and immune function. *The Journal of Pharmacology and Experimental Therapeutics*.

[27] Spittler A, Razenberger M, Kupper H (2000). Relationship between interleukin-6 plasma concentration in patients with sepsis, monocyte phenotype, monocyte phagocytic properties, and cytokine production. *Clinical Infectious Diseases*.

[28] Morse D, Pischke SE, Zhou Z (2003). Suppression of inflammatory cytokine production by carbon monoxide involves the JNK pathway and AP-1. * The Journal of Biological Chemistry*.

[29] Moore BA, Otterbein LE, Türler A, Choi AMK, Bauer AJ (2003). Inhaled carbon monoxide suppresses the development of postoperative ileus in the murine small intestine. *Gastroenterology*.

[30] Wagener FADTG, Da Silva J-L, Farley T, De Witte T, Kappas A, Abraham NG (1999). Differential effects of heme oxygenase isoforms on heme mediation of endothelial intracellular adhesion molecule expression. *The Journal of Pharmacology and Experimental Therapeutics*.

[31] Nakao A, Kimizuka K, Stolz DB (2003). Carbon monoxide inhalation protects rat intestinal grafts from ischemia/reperfusion injury. *The American Journal of Pathology*.

[32] Neto JS, Nakao A, Kimizuka K (2004). Protection of transplant-induced renal ischemia-reperfusion injury with carbon monoxide. * American Journal of Physiology: Renal Physiology*.

[33] Uchiyama K, Naito Y, Takagi T (2010). Carbon monoxide enhance colonic epithelial restitution via FGF15 derived from colonic myofibroblasts. *Biochemical and Biophysical Research Communications*.

[34] Naito Y, Takagi T, Uchiyama K, Yoshikawa T (2011). Heme oxygenase-1: a novel therapeutic target for gastrointestinal diseases. *Journal of Clinical Biochemistry and Nutrition*.

[35] Xia Y, Rao J, Yao A, Zhang F, Li G, Wang X (2012). Lithium exacerbates hepatic ischemia/reperfusion injury by inhibiting GSK-3beta/NF-kappaB-mediated protective signaling in mice. *European Journal of Pharmacology*.

[36] Platzer C, Fritsch E, Elsner T, Lehmann MH, Volk HD, Prosch S (1999). Cyclic adenosine monophosphate-responsive elements are involved in the transcriptional activation of the human IL-10 gene in monocytic cells. *European Journal of Immunology*.

[37] Hu X, Paik PK, Chen J (2006). IFN-*γ* Suppresses IL-10 production and synergizes with TLR2 by regulating GSK3 and CREB/AP-1 Proteins. *Immunity*.

[38] Song Y-A, Park Y-L, Kim K-Y (2011). Black tea extract prevents lipopolysaccharide-induced NF-*κ*B signaling and attenuates dextran sulfate sodium-induced experimental colitis. *BMC Complementary and Alternative Medicine*.

[39] Villegas I, La Casa C, Orjales A, Alarcon de la Lastra C (2003). Effects of dosmalfate, a new cytoprotective agent, on acute and chronic trinitrobenzene sulphonic acid-induced colitis in rats. *European Journal of Pharmacology*.

[40] Takagi T, Naito Y, Mizushima K (2010). Inhalation of carbon monoxide ameliorates TNBS-induced colitis in mice through the inhibition of TNF-*α* expression. *Digestive Diseases and Sciences*.

[41] Sugimoto K, Ogawa A, Mizoguchi E (2008). IL-22 ameliorates intestinal inflammation in a mouse model of ulcerative colitis. *The Journal of Clinical Investigation*.

[42] Berg DJ, Davidson N, Kühn R (1996). Enterocolitis and colon cancer in interleukin-10-deficient mice are associated with aberrant cytokine production and CD4(+) Th1-like responses. *The Journal of Clinical Investigation*.

[43] Dugo L, Collin M, Allen DA (2005). GSK-3*β* inhibitors attenuate the organ injury/dysfunction caused by endotoxemia in the rat. *Critical Care Medicine*.

[44] Cohen P, Frame S (2001). The renaissance of GSK3. *Nature Reviews Molecular Cell Biology*.

[45] Hofmann C, Dunger N, Schölmerich J, Falk W, Obermeier F (2010). Glycogen synthase kinase 3-*β*: a master regulator of toll-like receptor-mediated chronic intestinal inflammation. *Inflammatory Bowel Diseases*.

